# NOT Gates Based on Protein Degradation as a Case Study for a New Modular Modeling via SBML Level 3—Comp Package

**DOI:** 10.3389/fbioe.2022.845240

**Published:** 2022-03-11

**Authors:** Biruck Woldai Abraha, Mario Andrea Marchisio

**Affiliations:** School of Pharmaceutical Science and Technology, Tianjin University, Tianjin, China

**Keywords:** SBML level 3, *S. cerevisiae*, ClpP-ClpX, protein degration, Boolean gates

## Abstract

In 2008, we were among the first to propose a method for the visual design and modular modeling of synthetic gene circuits, mimicking the way electronic circuits are realized *in silico*. Basic components were DNA sequences that could be composed, first, into transcription units (TUs) and, then, circuits by exchanging fluxes of molecules, such as PoPS (polymerase per second) and RiPS (ribosomes per seconds) as suggested by Drew Endy. However, it became clear soon that such fluxes were not measurable, which highlighted the limit of using some concepts from electronics to represent biological systems. SBML Level 3 with the *comp* package permitted us to revise circuit modularity, especially for the modeling of eukaryotic networks. By using the libSBML Python API, TUs—rather than single parts—are encoded in SBML Level 3 files that contain species, reactions, and *ports*, i.e., the interfaces that permit to wire TUs into circuits. A circuit model consists of a collection of SBML Level 3 files associated with the different TUs plus a “main” file that delineates the circuit structure. Within this framework, there is no more need for any flux of molecules. Here, we present the SBML Level 3-based models and the wet-lab implementations of Boolean NOT gates that make use, in the yeast *Saccharomyces cerevisiae*, of the bacterial ClpX-ClpP system for protein degradation. This work is the starting point towards a new piece of software for the modular design of eukaryotic gene circuits and shows an alternative way to build genetic Boolean gates.

## Introduction

Like electronics, Synthetic Biology deals with circuits. Thus, over the last twenty years, efforts have been made to adapt electrical engineering concepts and methods to the modular design and modeling of circuits made of DNA. In his famous 2005 paper (Endy, 2005), Drew Endy depicted a possible correspondence between the electrical current—easily measurable with an ammeter—and biological currents that could be responsible for the working of synthetic gene circuits. Following the dogma of molecular biology, RNA polymerases and ribosomes, which lead the synthesis of mRNA and proteins, respectively, appeared to be the biological counterparts of the electrons. Moreover, basic circuit components (in eukaryotic cells) were identified with promoters, coding regions (CDSs), and terminators, i.e., DNA pieces with a well-defined function either in transcription or translation. As the electric current permits to wire together resistors, batteries, solenoids and all the other basic electric components, the fluxes of RNA polymerase (PoPS: polymerases per second) and ribosomes (RiPS: ribosomes per second) should be the biological currents (a shared input/output) that permit to assemble together, first, biological parts into transcription units (TUs) and then TUs into circuits. Hence, biological fluxes permitted to define concepts such as *part composability* and *abstraction hierarchy* in Synthetic Biology.

Computational biologists liked these innovatory ideas because they indicated how to deal with biological circuits in a systematic way, whereas wet-lab biologists were skeptical since they were well-aware of the fact that a disordered motion like that of RNA polymerases and ribosomes could not be measured with any instruments. Therefore, PoPS/RiPS-based modeling was never completely accepted by the Synthetic Biology community and, in the end, it showed—in our opinion—the limits of using electronics concepts to study biological systems.

We were among the first to develop a piece of software (Marchisio and Stelling, 2008)—later named “Parts and Pools” (Marchisio, 2014b)—based on an extension of the ideas of Drew Endy. We pointed out that PoPS and RiPS allowed, indeed, parts’ composability but were not enough to describe the wiring among transcription units, which is what really makes a circuit work. Therefore, we had to introduce other *signal carriers* (transcription factors, small RNAs, and chemicals) and their corresponding fluxes to mediate the interaction among TUs. Our piece of software was an add-on of ProMoT (process modeling tool) (Mirschel et al., 2009), a program for the visual, modular design of complex systems. ProMoT demands to program in MDL (model definition language), a Lisp-based language that permits to define modules that communicate via fluxes (of molecules, in biological systems) calculated and exchanged via the so-called module *terminals*. ProMoT provides a Graphical User Interface as well where modules are displayed in a drag-and-drop way and terminals are wired together.

Our ProMoT add-on is a collection of Perl and Python scripts each generating one or more MDL modules. The latest version (Marchisio et al., 2013) was successfully applied to the design and modeling of eukaryotic circuits. The usage of fluxes made it straightforward to build models based on mass-action kinetics. Moreover, eukaryotic promoters and coding regions can be characterized by a high number of species and reactions that are calculated by means of BioNeTGen (Blinov et al., 2004). The CDS script generates not only the MDL file for the CDS part, but also those corresponding to the pools of its mRNA and protein. Nucleus and cytoplasm are designed separately (due to their complexity) and then connected by running another script. Finally, a circuit is encoded by a collection of MDL files associated with parts and pools plus a main one that contains the connections among all the modules. ProMoT is able to export the circuit into the SBML2 format that permits circuit analysis and simulations—with, for instance, COPASI (Hoops et al., 2006) as we generally did—but loses the circuit modularity.

SBML Level 3 (Keating et al., 2020) with the hierarchical model composition package (*comp*) (Smith et al., 2015) allows for a direct modular modeling of genetic circuit without the need for the help of another language, such as MDL, nor the usage of fluxes of molecules to establish the wiring among the modules present in the circuit. Furthermore, SBML Level 3-comp (concisely SBML3-comp) files can be easily generated by means of the libSBML (Bornstein et al., 2008).

In this paper we describe the wet-lab implementation, in the yeast *Saccharomyces cerevisiae*, of two NOT gates based on protein degradation—a design strategy advanced 15 years ago (Grilly et al., 2007) but never extensively exploited nor optimized—and then we show how they can be modeled, in a modular way, by means of SBML3-comp. We will not explain all the libSBML commands required to generate the full circuit model (for this we refer the reader to our book chapter (Marchisio, 2021) and the files available at GitHub—see Data Availability below) but we will focus on the way to establish connections among circuit modules, which no longer demands to define fluxes of molecules that cannot be measured in the lab. SBML3-comp is already supported by several computational tools such as COPASI, iBioSim (Watanabe et al., 2019), Virtual Part Repository 2 (Misirli et al., 2021), Tellerium (Choi et al., 2018), and BMSS (Yeoh et al., 2019). For this reason, we think it will become the standard language to model genetic circuit in the very next future.

## Materials and Methods

### Wet-Lab Experiments

Backbones for all plasmids constructed in this work were the yeast integrative shuttle-vector pRSII40X available at Addgene (a gift from Steven Haase) (Chee and Haase, 2012). The plasmids containing ClpP, the yeast codon-optimized version of ClpX, and yEGFP_ssrA were kindly provided by Jeff Hasty (University of California, San Diego, United States). Each new plasmid was assembled via the Gibson method (Gibson et al., 2009)—they are listed in Supplementary Table S5. The DNA sequences of the DNA parts used in this work are written in Supplementary Material as well. Plasmids were integrated into the genome of the yeast *S. cerevisiae* strain CEN.PK2-1C (MATa; his3D1; leu2-3_112; ura3-52; trp1-289; MAL2-8c; SUC2)—Euroscarf (Johann Wolfgang Goethe University, Frankfurt, Germany)—via the lithium-acetate protocol (Gietz and Woods, 2002).

Green fluorescence was measured with a BD FACSVerse™ Flow Cytometer (blue laser-488 nm, emission filter-527/32 nm). Each strain was measured in three replicas. During each experiment, 30,000 events were recorded. Data from the flow cytometer was analyzed with the flowcore R-Bioconductor package (Hahne et al., 2009).

The performance of a Boolean gate was characterized by the ON/OFF ratio, i.e., the the ratio between the maximal (ON) and minimal (OFF) fluorescence level expressed by the circuit. For many applications, an ON/OFF ratio bigger than or equal to 2 is enough to claim that a Boolean gate works properly (Yu and Marchisio, 2021; Zhang and Marchisio, 2022).

### Computational Experiments

Scripts to generate circuit modules were written in Python (version 3.8) and run on a MacBook Air (macOS Catalina 10.15.7, 1.6 GHz Dual-Core Intel Core i5, 8 GB RAM). Circuit simulations and analysis were carried out with COPASI (version 4.34, build 251).

## Results

In *E. coli*, the ClpX and ClpP proteins work in tandem (ClpXP) in order to carry out protein degradation. Upon recognition of a degron sequence, ClpX proceeds to unfold and translocate a protein into the ClpP proteolytic compartment with the release of small peptides (Baker and Sauer, 2012). This bacterial system was first shown to work efficiently in *S. cerevisiae* by Grilly and co-authors (Grilly et al., 2007)—and later re-proposed by Macia *et al.* (Macia et al., 2016)—who constructed a four-gene circuit where the yeast enhanced green fluorescent protein—yEGFP (Sheff and Thorn, 2004)—was fused to the ssrA degradation tag and the expression of both ClpX and ClpP was controlled by the LacI/IPTG system. Moreover, yEGFP was produced only in the presence of galactose in the cell culture (see Supplementary Figure S1). Taking a cue from this work, we made use of the ClpXP system to construct two different NOT gates, one responding to galactose, the other to beta-estradiol.

### Galactose-Responsive NOT Gate

**FIGURE 1:**
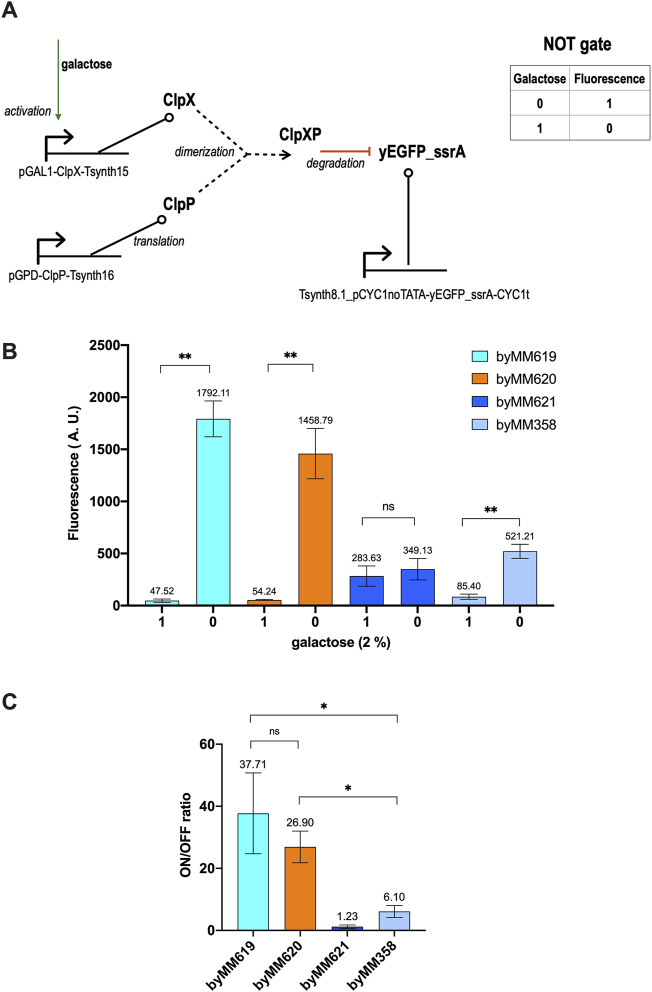
Galactose-sensing NOT gate. **(A)** Circuit scheme. The reporter protein (yEGFP_ssrA) and ClpP are constitutively expressed by the rather weak synthetic promoter Tsynth8.1_pCYC1noTATA and the strong yeast *GPD* promoter, respectively. ClpX (a yeast codon optimized version) synthesis is switched ON only in the presence of galactose. Differently from the network in (Grilly et al., 2007), only the expression of one of the two components of the ClpXP dimer is controlled by an input. Moreover, the production of the tagged green fluorescent protein cannot be stopped by any external signal (see Supplementary Figure S1 for a comparison). **(B)** Average fluorescence level (together with its standard deviation) corresponding to four strains hosting a NOT gate. byMM619 and byMM620 show the highest separation between the 0 and 1 output level. byMM621, in contrast, turned out to be unfunctional. byMM358 differs from the previous three gates for the synthetic promoter DEG1t_pCYC1noTATA that expresses yEGFP_ssrA instead of Tsynth8.1_pCYC1noTATA. DEG1t_pCYC1noTATA is 1.37-fold stronger than Tsynth8.1_pCYC1noTATA. **(C)** Gate performance in term of fluorescence gain. byMM619 and byMM620 turned out not to be significantly different under statistical analysis. Their mean gain is much higher than that of byMM358, which is nevertheless reasonably elevated for a logic circuit. Each experiment was made in three replicas. Data has been analyzed via two-sided Welch’s *t*-test (*: *p*-value < 0.05; **: *p*-value < 0.01; ns: no significant difference).

The galactose responsive NOT gate requires three transcription units only. We implemented two versions of it by changing the synthetic constitutive promoter upstream of *yEGFP_ssrA*: Tsynth8.1_pCYC1noTATA and DEG1t_pCYC1noTATA (Song et al., 2016). The former configuration returned the best results with an around 30-fold ON/OFF ratio (see Figure 1).

#### Modular Modeling With SBML3-Comp

The circuit scheme in Figure 1A shows only the DNA sequences required to build the gate and the proteins that make the circuit work. Several important features are, however, missing such as the cell compartments and the mRNA corresponding to each protein. By using SBML3-comp, we made a model for this NOT gate that consists of 16 species and 23 reactions distributed over 7 modules: 3 TUs (in the nucleus), 3 mRNA pools, and a *degradation* pool where ClpP and ClpX first dimerize and then degrade, quickly, yEGFP_ssrA. All pools lie in the cytoplasm since none of the proteins involved in the circuit interacts with the DNA.

#### Connecting Modules via Ports

**FIGURE 2:**
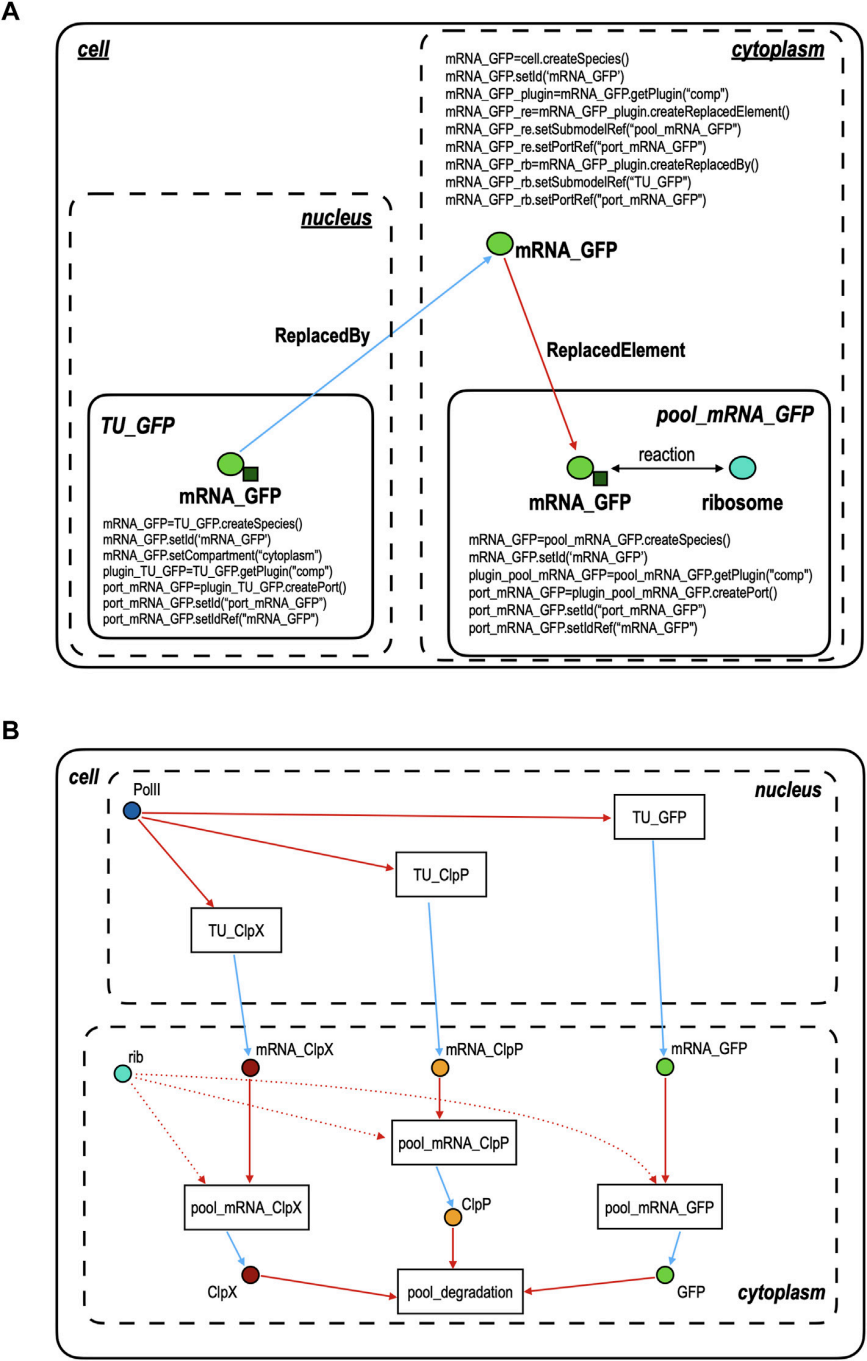
Connecting modules in SBML3-comp. **(A)** Port objects (represented as small dark green squares) are instantiated in the TU_GFP and pool_mRNA_GFP modules and refer to the species mRNA_GFP (a light green circle)—a copy of which is present in each of the two modules. Ports permit to link the two modules indirectly, i.e., via a third copy of mRNA_GFP that lies in the cytoplasm (cell module) and is connected to the mRNA_GFP inside TU_GFP via a ReplacedBy object (cyan arrow) and to the mRNA_GFP inside pool_mRNA_GFP via a ReplacedElement object (red arrow). **(B)** Modular model of the galactose responding NOT gate shown in Figure 1A. Species, like mRNA_GFP, instantiated in the cell model are responsible for the connection between at least two modules. As in Figure 2A, all cyan arrows represent a ReplacedBy relation and the red arrows (straight or dotted) a ReplacedElement one. Galactose, the circuit input, is not shown since its presence or absence is determined by changing the value of the transcription initiation rate of pGAL1, which belongs to the module TU_ClpX. It should be noted that the circuit design still follows the idea to have two main kinds of module, like in our piece of software “Parts and Pools”. However, by using SBML3-comp, some Pools become redundant and are replaced by single species, whereas Parts are no longer modeled separately because, as explained in the main text, TUs represent now the smallest circuit components. This assumption permits to remove several species (compounds) and reactions that were necessary to describe the interactions between RNA polymerases II and the DNA Parts—and also between the ribosomes and the mRNA segments.

The main feature we want to explain about modeling gene circuits in SBML3-comp is how to realize the connection between two (or more) modules without the need for fluxes. SBML3-comp permits to associate species with *ports*. They are objects that have the function to establish a link among *copies* of the same species that lie in different modules. However, ports cannot be connected directly but they need a “helper” species, i.e., a third copy of the same species that lies, however, in a *compartment* (or another module). This helper species does not have its own port but has the capability to finalize the junction between two ports. Figure 2A clarifies how ports, species, and modules work together by showing how the mRNA corresponding to yEGFP_ssrA (mRNA_GFP) is the species that permits to link the transcription unit encoding for yEGFP_ssrA in the nucleus (TU_GFP) to the pool, in the cytoplasm, where yEGFP_ssrA is synthesized (pool_mRNA_GFP). In this representation, we have three kinds of modules: the cell, which represents a *model* in SBML3-comp, TU_GFP and pool_mRNA_GFP, which are both *submodels* of the cell model, and the compartments (the nucleus and the cytoplasm). A model is a global container of submodels and compartments. TU_GFP is included in the nucleus and, from Figure 2B, we can see that it gets RNA polymerase II (PolII) as an input and delivers mRNA_GFP as an output. mRNA_GFP is created, as a species, in each of the three modules where it is contained, i.e., TU_GFP, the cytoplasm, and pool_mRNA_GFP. It should be noted, however, that the TU_GFP code contains the line “mRNA_GFP.setCompartment (“cytoplasm”)” which means that, even though the module lies in the nucleus, its output will stay in the cytoplasm. Every module gets access to the package comp via the command “getPlugin (“comp”)”, that permits the creation of ports in the submodels and references to species in the main model. In our example, we have that the submodel TU_GFP instantiates a new “comp” object—here called “plugin_TU_GFP”—that allows the creation of a port object via the instruction port_mRNA_GFP = plugin_TU_GFP.createPort(). The new port is then given an ID (i.e. a name: port_mRNA_GFP.setId (“port_mRNA_GFP”) and is associated with the mRNA_GFP species through the command: port_mRNA_GFP.setIdRef(“mRNA_GFP”). By using the same syntax, a port named “port_mRNA_GFP” that refers to the species mRNA_GFP is generated also in the other submodel, pool_mRNA_GFP. These two ports are linked thanks to the mRNA_GFP species created in the cell model. Here, no new ports are made. mRNA_GFP invokes the getPlugin (“comp”) command to establish two new objects. One belongs to the *ReplacedBy* class and sets up a bond with the TU_GFP submodel via the local port_mRNA_GFP. The other is a *ReplacedElement* object that “points” to the port_mRNA_GFP in the submodel pool_mRNA_GFP. In this way, a connection among the three mRNA_GFP species is established. As shown in Figure 2B, 5 more “helper” species are present in the cell model to realize connections between pairs of modules. The species “PolII” and “rib” do not belong to any submodel. Thus, our SBML3-comp model does not contain the RNA polymerase II and the ribosome pools. These species are linked to TUs and pool_mRNAs simply via ReplacedElement objects. Every submodel plus the cell model is saved as a separate xml file. In order to simulate the circuit with COPASI, it is enough to import the “cell.xml” file that calls all the other xml files and *flattens* the overall model such that, for instance, only one species called mRNA_GFP is present and lies in the cytoplasm.

#### Comparing Computational and Experimental Results

The mathematical model for the whole gate assumes that promoters and mRNAs are activated by RNA polymerases II and ribosomes, respectively, via a Hill function without cooperativity. The ClpXP complex is supposed to bind irreversibly to yEGFP_ssrA and induce its fast degradation. Parameters values were taken, where possible, from our previous works (Marchisio, 2014a; 2021), estimated from lab measurements, and optimized with COPASI to reach an ON/OFF ratio equal to 29.56, i.e. in reasonable agreement with our experimental results. A detailed description of the model of the galactose-sensing NOT gate is given in the Supplementary Model 1.

### Beta-Estradiol Responsive NOT Gate

**FIGURE 3:**
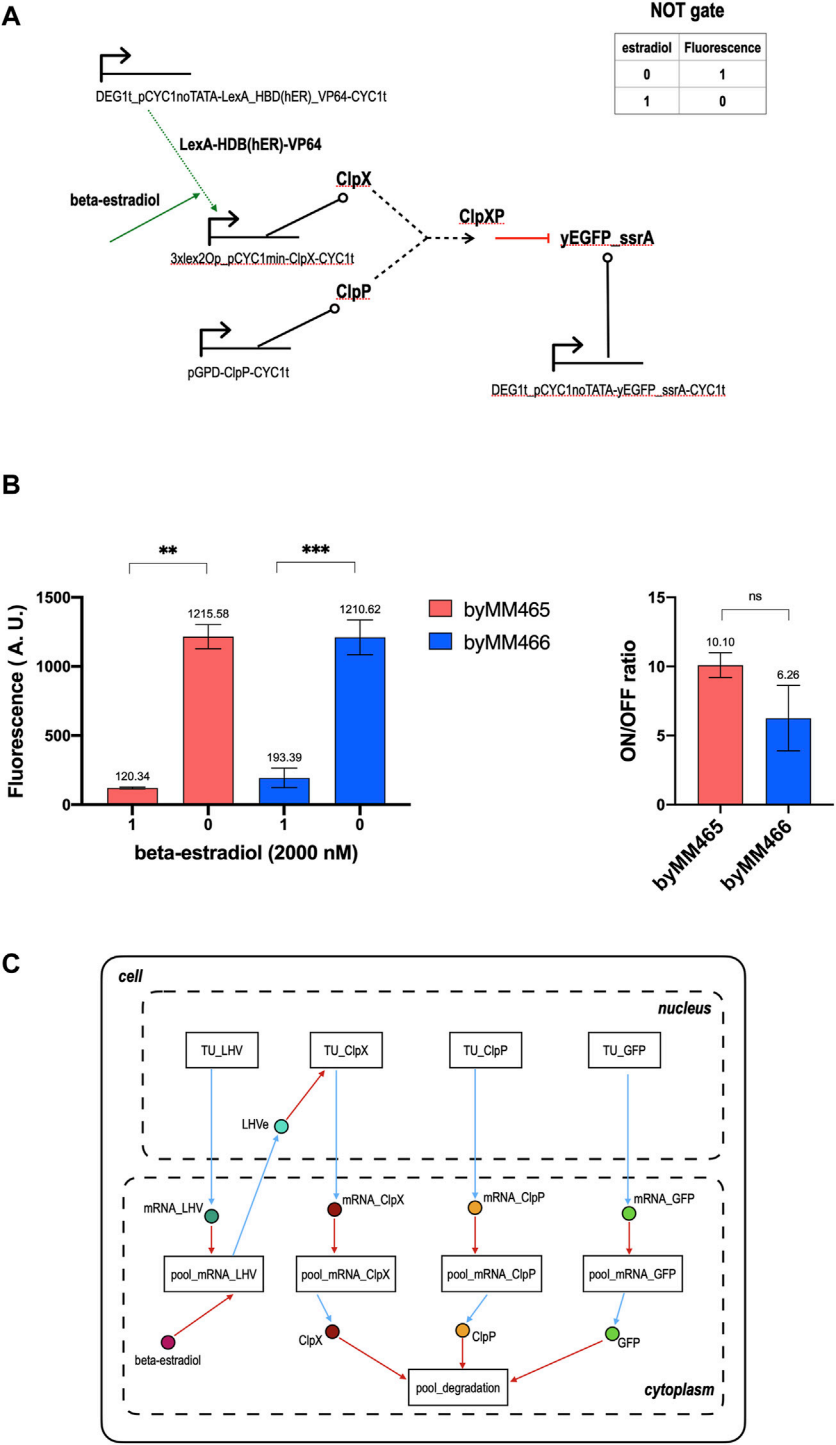
NOT gate sensing beta-estradiol. **(A)** Circuit scheme. The synthesis of ClpX depends on the presence of the hormone beta-estradiol, the gate input. The additional TU with respect to the gate in Figure 1 synthesizes, constitutively, a chimeric activator that, in our model, we called LHV since it consists of the bacterial protein LexA (acting as a DNA-binding domain), the hormone-binding domain of the human estrogen receptor (HBD(ER)), and the viral strong activation domain VP64. In the absence of beta-estradiol, LHV is kept in the cytoplasm because of the action of Hsp90 that binds HBD(hER). As a consequence, the synthetic activated promoter upstream of ClpX, which is made of three copies of lex2Op (the LexA operator) in front of a minimal weak *CYC1* promoter (Ottoz et al., 2014), is incapable of producing ClpX in high quantity such that the gate fluorescent output is high. A concentration of 1-2 µM beta-estradiol, which bind HBD (hER), nullifies the effect of Hsp90 and lets the complex LHVe (LexA_HBD(hER)_VP64-beta-estradiol) get into the nucleus and enhance the synthesis of ClpX, upon binding the three lex2Ops upstream of pCYC1min. Under these conditions, ClpX is highly expressed and, as a consequence, the fluorescence emitted by the gate drops to a very low level. **(B)** Mean fluorescence level and ON/OFF ratio of our two NOT gate implementations responsive to beta-estradiol. Every value corresponds to the mean of three replicas. The standard deviation of the mean is shown as well. Data has been analyzed via two-sided Welch’s *t*-test (**: *p*-value < 0.01; ***: *p*-value < 0.001; ns: no significant difference). **(C)** NOT gate modular modeling. The gate input is now a species in the cell cytoplasm connected, via a ReplacedElement object, to the pool_mRNA_LHV, where it binds the chimeric activator LHV and forms the complex LHVe. This species lies in the nucleus and permits a direct connection between pool_mRNA_LHV and TU_ClpX. These two modules contain all the reactions where LHVe is involved in.

With respect to the galactose-sensing NOT gate, the scheme of that responding to beta-estradiol demands one more TU encoding for a chimeric activator that interacts with the circuit input (see Figure 3A). In the absence of beta-estradiol, ClpX is expressed at a very low quantity and, consequently, the fluorescence level of the circuit is high. In contrast, concentrations of beta-estradiol between 1 and 2 µM trigger an elevated production of ClpX that induces a drop in the output signal. Our two implementations gave an ON/OFF ratio equal to 10.10 and 6.26. However, they did not show any significant statistical difference (see Figure 3B and Supplementary Figures S1, S2 for the corresponding titration curves). We modeled this circuit in a slightly different way with respect to the previous one, i.e. we neglected the presence of RNA polymerase II and ribosomes in the cells—a commonly used simplification that fully excludes fluxes such as PoPS and RiPS. Overall, we needed 9 modules (4 TUs in the nucleus, 4 mRNA pools and one degradation pool in the cytoplasm) and as many “connecting” species instantiated in the cell model: 8 in the cytoplasm and only one in the nucleus (see Figure 3C). On the whole, the model consists of 21 species and 35 reactions. After parameter optimization, we got a simulated ON/OFF ratio equal to 13.64, not too distant from the measured one (more details are given in Supplementary Model 2).

## Discussion

SBML3-comp represents a huge improvement of SBML2 since it permits to construct models of biological systems and, therefore, of synthetic gene circuits, in a modular way without being assisted by other programs. libSBML permits to divide the model of a circuit in different scripts, each generating one or more modules. We think that the best way is to treat transcription units, rather than single DNA parts, as basic modules. A TU can be encoded in the same file together with its corresponding pool of mRNA and, if required, that of the protein it encodes for. The direct use of SBML3 allows also to choose easily the best kinetics for every reaction, without the constraint of using the same in the whole circuit.

In this work we have shown how Boolean gates characterized by high ON/OFF ratio can be built, in *S. cerevisiae*, by using an almost forgotten, though powerful, yeast-orthogonal system, i.e., the bacterial ClpXP dimer to induce protein degradation. Our Boolean gates served as a case study to explain how to use SBML3-comp to model genetic circuits in a new modular way. Importantly, it was apparent how Python scripts, which generate the SBML3-comp files, can be recycled for the same TUs inside different circuits or just slightly modified to construct models for similar TUs.

We think that the joint use of the System Biology Open Language—SBOL (Galdzicki et al., 2014) and SBML3-comp seems to be the best way to combine, in the future, a detailed description of the actual DNA circuit components (sequences) with modular modeling (Watanabe et al., 2019; Misirli et al., 2021).

## Data Availability

FACS data (fcs files) relating to the results presented in this work are accessible at FlowRepository (http://flowrepository.org). Python files to generate the model of the two different NOT gates, together with COPASI files containing the optimized parameter values, are publicly available at GitHub (https://github.com/mamarchisio/ClpXP-Python-COPASI).
