# Supplementary material for: NOT Gates Based on Protein Degradation as a Case Study for a New Modular Modeling via SBML Level 3—Comp Package
Source: Front Bioeng Biotechnol. 2022 Mar 11;10:845240. doi: 10.3389/fbioe.2022.845240 (PMC8961978; doi:10.3389/fbioe.2022.845240)
Supplement: Supplementary file 1 [file DataSheet1.PDF]

NOT gates based on protein degradation as a case study for a new modular modeling via SBML Level 3 - comp package.
Supplementary Material

Biruck Woldai Abraha[1] and Mario Andrea Marchisio[1,*]

[1]School of Pharmaceutical Science and Technology, Tianjin University,

92 Weijin Road, 300072-Tianjin, China

[*]corresponding author; emails: mario@tju.edu.cn, mamarchisio@yahoo.com

Model 1: galactose-sensing NOT gate

In order to use the data from FACS experiments when running parameter optimization, we considered that each fluorescent protein contributed with a single arbitrary unit of fluorescence.

Species present in the model

- $pGAL1$, the yeast galactose-inducible strong promoter
- pm_ClpX, pre-mature mRNA of ClpX
- $mRNA\_ClpX$
- $ClpX$
- $pGPD$, the yeast constitutive strong promoter
- pm_ClpP, pre-mature mRNA of ClpP
- $mRNA\_ClpP$
- $ClpP$
- $pTs8$, the synthetic promoter Tsynth8.1_pCYC1noTATA
- pm_GFP, pre-mature mRNA of yEGFP_ssrA
- $mRNA\_GFP$
- GFP, corresponds to yEGFP_ssrA
- $ClpXP$, the ClpX-ClpP complex
- $ClpXP\_GFP$, the ClpXP-yEGFP_ssrA complex
- $PolII$, RNA polymerase II
- rib, stands for ribosome

Reactions considered in the model

$$PolII + pGAL1 \xrightarrow{\mathrm{f}(k_{2\mathrm{pGAL1}}, K_{HPolII})} PolII + pGAL1 + pm\_ClpX$$
$$pm\_ClpX \xrightarrow{k_{\mathrm{mt}}} mRNA\_ClpX$$
$$pm\_ClpX \xrightarrow{k_{\mathrm{d}}}$$

$$PolII + pGPD \xrightarrow{\text{f}(k_{\text{2pGPD}},K_{\text{HPolII}})} PolII + pGPD + pm\_ClpP$$
$$pm\_ClpP \xrightarrow{k_{\text{mt}}} mRNA\_ClpP$$
$$pm\_ClpP \xrightarrow{k_{\text{d}}}$$
$$PolII + pTs8 \xrightarrow{\text{f}(k_{\text{2pTs8}},K_{\text{HpolII}})} PolII + pTs8 + pm\_GFP$$
$$pm\_GFP \xrightarrow{k_{\text{mt}}} mRNA\_GFP$$
$$pm\_GFP \xrightarrow{k_{\text{d}}}$$
$$rib + mRNA\_ClpX \xrightarrow{\text{f}(k_{\text{2r}},K_{\text{Hrib}})} rib + mRNA\_ClpX + ClpX$$
$$mRNA\_ClpX \xrightarrow{k_{\text{d}}}$$
$$rib + mRNA\_ClpP \xrightarrow{\text{f}(k_{\text{2r}},K_{\text{Hrib}})} rib + mRNA\_ClpP + ClpP$$
$$mRNA\_ClpP \xrightarrow{k_{\text{d}}}$$
$$rib + mRNA\_GFP \xrightarrow{\text{f}(k_{\text{2r}},K_{\text{Hrib}})} rib + mRNA\_GFP + GFP$$
$$mRNA\_GFP \xrightarrow{k_{\text{d}}}$$
$$ClpX + ClpP \xrightarrow{k_1} ClpXP$$
$$ClpXP \xrightarrow{k_{-1}} ClpX + ClpP$$
$$ClpX \xrightarrow{k_{\text{dp}}}$$
$$ClpP \xrightarrow{k_{\text{dp}}}$$
$$ClpXP \xrightarrow{k_{\text{dp}}}$$
$$GFP \xrightarrow{k_{\text{dg}}}$$
$$ClpXP + GFP \xrightarrow{k_c} ClpXP\_GFP$$
$$ClpXP\_GFP \xrightarrow{k_{df}} ClpXP$$

It should be noted that the notation f(k_{2A},K_{HB}) (where A and B are two species) corresponds to

$$f(k_{2A}, K_{HB}) = k_{2A} \cdot A \cdot \frac{\frac{B}{K_{HB}}}{1 + \frac{B}{K_{HB}}}$$

Table S1: Model parameters

Quantity	Value	Unit	Meaning and reference
PolII	2000	-	number of molecules; tuned [1]
rib	5000	-	number of molecules; tuned [1]
pGAL1	1	-	number of molecules
pGPD	1	-	number of molecules
pTs8	1	-	number of molecules
Nucleus - volume	$2.9\,10^{-15}$	l	- [1]
Cytoplasm - volume	$3.9\,10^{-14}$	l	- [1]
k_{2pGAL1}	0.46	s^{-1}	transcription initiation rate; optimized
$K_{HpPolII}$(pGAL1)	$8.75\,10^{-7}$	M	Half-saturation constant; optimized
k_{mt}	$5.5\,10^{-4}$	s^{-1}	mRNA maturation and translocation rate (corresponds to 30 min.) [2]
k_d	$5.7\,10^{-4}$	s^{-1}	pre-mRNA decay rate (corresponds to 20 min.) [2]
k_{2pGPD}	0.59	s^{-1}	transcription initiation rate; optimized
K_{HPolII}(pGPD)	$1.55\,10^{-11}$	M	Half-saturation constant; optimized
k_{2pTs8}	0.21	s^{-1}	transcription initiation rate; optimized
K_{HPolII}(pTs8)	$9.71\,10^{-6}$	M	Half-saturation constant; optimized
k_{2r}	0.01	s^{-1}	translation initiation rate; tuned [1]
K_{Hrib}	10^{-7}	M	Half-saturation constant; [2]
k_1	$1.30\,10^{5}$	$M^{-1}s^{-1}$	ClpXP formation rate-constant; optimized
k_{-1}	0.71	s^{-1}	ClpXP dissociation rate; optimized
k_{dp}	$2.7\,10^{-4}$	s^{-1}	protein decay rate (corresponds to 40 min.) [1]
k_{dg}	$8.25\,10^{-05}$	s^{-1}	GFP decay rate (corresponds to a dilution time of 140 min.) [2]
k_c	$1.30\,10^{5}$	$M^{-1}s^{-1}$	ClpXP - GFP association rate constant; optimized
k_{df}	0.0023	s^{-1}	fast GFP decay rate (corresponds to 2 min.) [2]

Results

Table S2: Truth table

Galactose	Number of molecules (GFP)
0	1478.16
1 (2%)	50.01

ON/OFF ratio: 29.56

Model 2: beta-estradiol-sensing NOT gate

Species present in the model

- $pDg1$, the synthetic constitutive promoter DEG1t_pCYC1noTATA. It drives the synthesis of LHV and GFP
- pm_LHV, pre-mature mRNA of LexA-HEB(hER)-VP64
- $mRNA\_LHV$
- LHV, LexA-HBD(hER)-VP64
- $LHVe\_c$, LexA-HBD(hER)-VP64 bound to beta estradiol, located in the cytoplasm
- $LHVe$, LexA-HBD(hER)-VP64 bound to beta estradiol, located in the nucleus
- $psCYC1$, the synthetic activated promoter 3xlex2Op_pCYC1min
- pm_ClpX, pre-mature mRNA of ClpX
- $mRNA\_ClpX$
- $ClpX$
- $pGPD$, the yeast constitutive strong promoter
- pm_ClpP, pre-mature mRNA of ClpP
- $mRNA\_ClpP$
- $ClpP$
- pm_GFP, pre-mature mRNA of yEGFP_ssrA
- $mRNA\_GFP$
- GFP, corresponds to yEGFP_ssrA
- $ClpXP$, the ClpX-ClpP complex
- $ClpXP\_GFP$, the ClpXP-yEGFP_ssrA complex
- *be*, beta-estradiol

Reactions considered in the model

$$pDg1 \xrightarrow{k_{\mathrm{2pDg1}}} pDg1 + pm\_LHV$$
$$pm\_LHV \xrightarrow{k_{\mathrm{mt}}} mRNA\_LHV$$
$$pm\_LHV \xrightarrow{k_{\mathrm{d}}}$$
$$mRNA\_LHV \xrightarrow{k_{\mathrm{2r}}} mRNA\_LHV + LHV$$
$$mRNA\_LHV \xrightarrow{k_{\mathrm{d}}}$$
$$be + LHV \xrightarrow{k_{\mathrm{b}}} LHVe\_c$$
$$LHVe\_c \xrightarrow{k_{\mathrm{u}}} be + LHV$$
$$LHVe\_c \xrightarrow{k_{\mathrm{t}}} LHVe$$
$$LHVe\_c \xrightarrow{k_{\mathrm{dp}}} be$$
$$LHV \xrightarrow{k_{\mathrm{dp}}}$$
$$LHVe + psCYC1 \xrightarrow{\mathrm{f}(k_{\mathrm{2psCYC1}}, K_{\mathrm{H\,LHVe}}, n_{\mathrm{H\,LHVe}})} LHVe + psCYC1 + pm\_ClpX$$
$$pm\_ClpX \xrightarrow{k_{\mathrm{mt}}} mRNA\_ClpX$$
$$pm\_ClpX \xrightarrow{k_{\mathrm{d}}}$$
$$LHVe \xrightarrow{k_{\mathrm{dp}}} \mathrm{be}$$
$$psCYC1 \xrightarrow{k_{\mathrm{2lk}}} psCYC1 + pm\_ClpX$$
$$mRNA\_ClpX \xrightarrow{k_{\mathrm{2r}}} + mRNA\_ClpX + ClpX$$
$$mRNA\_ClpX \xrightarrow{k_{\mathrm{d}}}$$
$$pGPD \xrightarrow{k_{\mathrm{2pGPD}}} pGPD + pm\_ClpP$$
$$pm\_ClpP \xrightarrow{k_{\mathrm{mt}}} mRNA\_ClpP$$
$$pm\_ClpP \xrightarrow{k_{\mathrm{d}}}$$
$$mRNA\_ClpP \xrightarrow{k_{\mathrm{2r}}} mRNA\_ClpP + ClpP$$
$$mRNA\_ClpP \xrightarrow{k_{\mathrm{d}}}$$
$$pDg1 \xrightarrow{k_{\mathrm{2pDg1}}} pDg1 + pm\_GFP$$
$$pm\_GFP \xrightarrow{k_{\mathrm{mt}}} mRNA\_GFP$$
$$pm\_GFP \xrightarrow{k_{\mathrm{d}}}$$
$$mRNA\_GFP \xrightarrow{k_{\mathrm{2r}}} mRNA\_GFP + GFP$$
$$mRNA\_GFP \xrightarrow{k_{\mathrm{d}}}$$
$$ClpX + ClpP \xrightarrow{k_{1}} ClpXP$$
$$ClpXP \xrightarrow{k_{-1}} ClpX + ClpP$$
$$ClpX \xrightarrow{k_{\mathrm{dp}}}$$

$$ClpP \xrightarrow{k_{\mathrm{dp}}}$$
$$ClpXP \xrightarrow{k_{\mathrm{dp}}}$$
$$GFP \xrightarrow{k_{\mathrm{dg}}}$$
$$ClpXP + GFP \xrightarrow{k_c} ClpXP\_GFP$$
$$ClpXP\_GFP \xrightarrow{k_{df}} ClpXP$$

Where it holds that

$$f(k_{2psCYC1}, K_{HLHVe}, n_{HLHVe}) = k_{2psCYC1} \cdot psCYC1 \cdot \frac{(\frac{LHVe}{K_{HLHVe}})^{n_{HLHVe}}}{1 + (\frac{LHVe}{K_{HLHVe}})^{n_{HLHVe}}}$$

Table S3: Model parameters

Quantity	Value	Unit	Meaning and reference
pDg1	2	-	number of molecules
pGPD	1	-	number of molecules
psCYC1	1	-	number of molecules
Nucleus - volume	$2.9\,10^{-15}$	l	- [1]
Cytoplasm - volume	$3.9\,10^{-14}$	l	- [1]
k_{2pDg1}	0.12	s^{-1}	transcription initiation rate; optimized
k_{mt}	$5.5\,10^{-4}$	s^{-1}	mRNA maturation and translocation rate (corresponds to 30 min.) [2]
k_d	$5.7\,10^{-4}$	s^{-1}	pre-mRNA decay rate (corresponds to 20 min.) [2]
k_{2r}	0.01	s^{-1}	translation initiation rate; tuned [1]
k_b	$3.94\,10^{7}$	$M^{-1}s^{-1}$	beta-estradiol - LHV binding rate; optimized
k_u	0.98	s^{-1}	LHVe_c dissociation rate; optimized
k_t	0.0083	s^{-1}	nucleic import rate (corresponds to 2 min.)[2]
k_{dp}	$2.7\,10^{-4}$	s^{-1}	protein decay rate (corresponds to 40 min.) [1]
$k_{2psCYC1}$	0.58	s^{-1}	transcription initiation rate; optimized
K_{HLHVe}	$8.75\,10^{-7}$	M	Half-saturation constant; optimized
n_{HLHVe}	1.52	-	Cooperativity coefficient; optimized
k_{2lk}	0.019	s^{-1}	transcription leakage rate of psCYC1 ; optimized
k_{2pGPD}	0.59	s^{-1}	transcription initiation rate; optimized
k_1	$1.30\,10^{5}$	$M^{-1}s^{-1}$	ClpXP formation rate-constant; optimized
k_{-1}	0.71	s^{-1}	ClpXP dissociation rate; optimized
k_{dg}	$8.25\,10^{-05}$	s^{-1}	GFP decay rate (corresponds to a dilution time of 140 min.) [2]
k_c	$1.30\,10^{5}$	$M^{-1}s^{-1}$	ClpXP - GFP association rate constant; optimized
k_{df}	0.0023	s^{-1}	fast GFP decay rate (corresponds to 2 min.) [2]

Results

Table S4: Truth table

Beta-estradiol	Number of molecules (GFP)
0	1457.91
1 $(2\mu M)$	106.86

ON/OFF ratio: 13.64

Figures

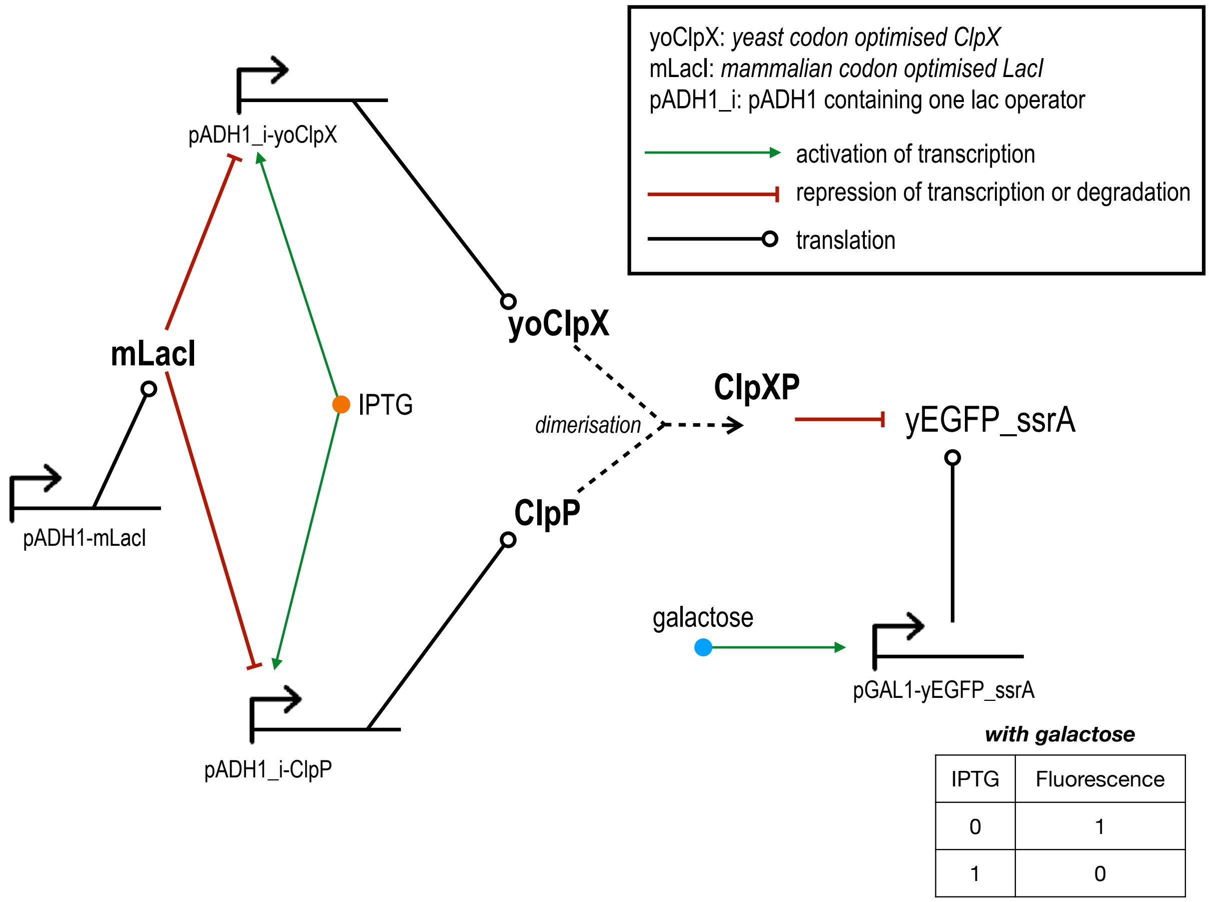

with galactose

IPTG	Fluorescence
0	1
1	0

Figure S1: ClpXP-based circuit by Grilly *et al.* [3]. Fluorescence expression demands galactose. The synthesis of both yoClpX and ClpP is repressed by mLacI unless IPTG is present in the cell culture.

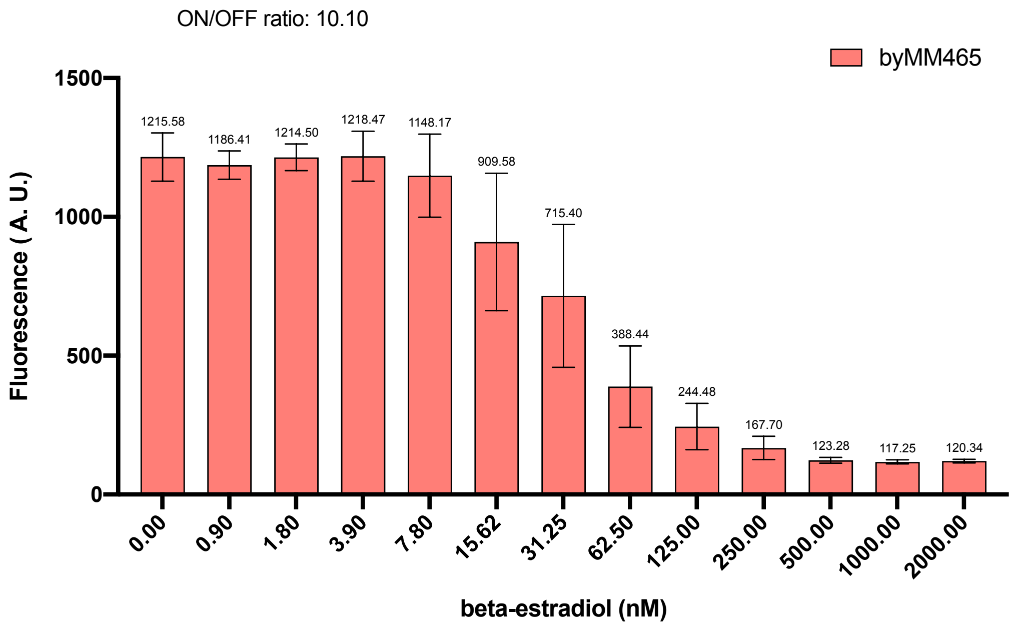

Figure S2: Beta-estradiol-responding NOT gate. Titration curve on byMM465 with beta-estradiol varying from 0 to 2 μM.

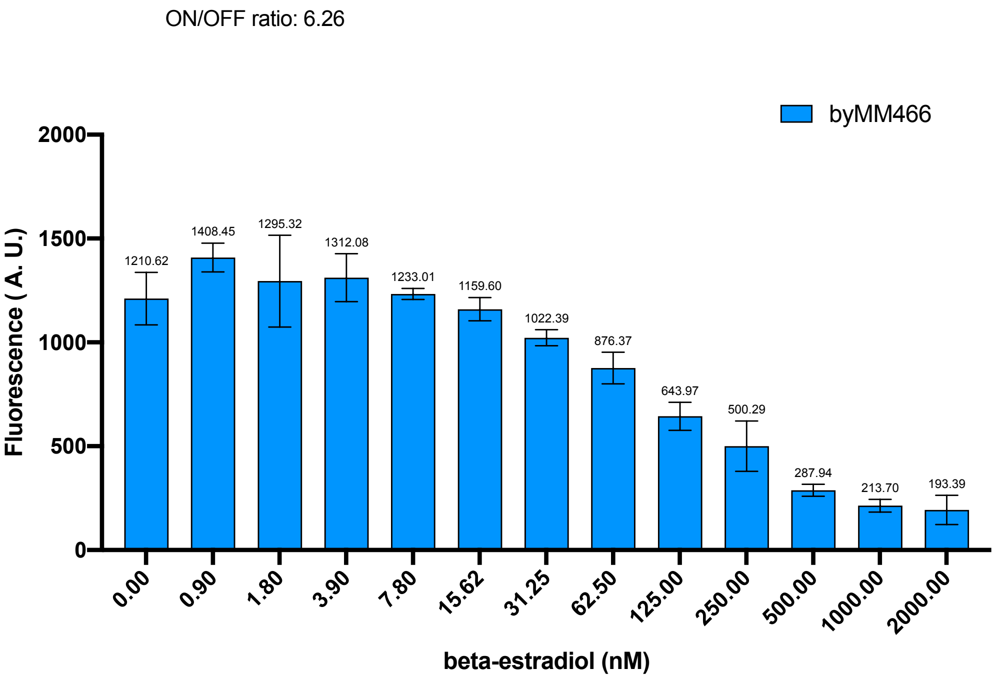

Figure S3: Beta-estradiol-responding NOT gate. Titration curve on byMM466 with beta-estradiol varying from 0 to $2\,\mu M$.

Plasmids realized in this work

Plasmid name	Construct
pMM767	pRSII403-Tsynth8.1_pCYC1noTATA-yEGFPgg-GS-ssrA-CYC1t
pMM777	pRSII405-pGAL1-HIStag-GS-yoClpX-Tsynth15
pMM785	pRSII406-pGPD-Flagtag-GS-ClpP-Tsynth16
pMM508	pRSII406-DEG1t_pCYC1noTATA-yEGFP_ssrA-CYC1t
pMM428	pRSII404-pGPD-ClpP-CYC1t
pMM530	pRSII405-pGAL1-yoClpX-CYC1t
pMM636	pRSII405-3xlex2Op_pCYC1min-yoClpX-CYC1t
pMM635	pRSII403-DEG1t_pCYC1noTATA-LexA-HBD(hER)-VP64-CYC1t

Table S5: List of the plasmids assembled in this work.

Notice: the BsaI site was removed from the original yEGFP sequence.
Tsynth15 and Tsynth16 have been taken from [4].

Yeast strains used/realized in this work

Strain name	Genotype
byMM584	CEN.PK2-1C (MATa; his3D1; leu2-3_112; ura3-52; trp1-289; MAL2-8c; SUC2)
byMM111	CEN.PK2-1C (MATa; his3D1; leu2-3_112; ura3-52; trp1-289; MAL2-8c; SUC2)
byMM589	byMM584 pMM763::HIS3
byMM606	byMM584 pMM763::HIS3 pMM777::URA3
byMM607	byMM584 pMM763::HIS3 pMM785::LEU2
byMM619	byMM584 pMM763::HIS3 pMM785::LEU2 pMM777::URA3
byMM620	byMM584 pMM763::HIS3 pMM785::LEU2 pMM777::URA3
byMM621	byMM584 pMM763::HIS3 pMM777::URA3 pMM785::LEU2
byMM311	byMM111 pMM508::URA3
byMM349	byMM111 pMM508::URA3 pMM428::TRP1
byMM358	byMM111 pMM508::URA3 pMM428::TRP1 pMM530::LEU2
byMM454	byMM111 pMM508::URA3 pMM428::TRP1 pMM636::LEU2
byMM455	byMM111 pMM508::URA3 pMM428::TRP1 pMM636::LEU2
byMM465	byMM111 pMM508::URA3 pMM428::TRP1 pMM636::LEU2 pMM635::HIS3
byMM466	byMM111 pMM508::URA3 pMM428::TRP1 pMM636::LEU2 pMM635::HIS3

Table S6: List of yeast strains realized in this work (plus the original one, CEN.PK2-1C). Plasmids are described in Tables S5.

DNA part sequences

Promoters

**Tsynth8.1_pCYC1 noTATA**	TTTCccTATATAAACTCATTTACTTATGTAGGAATAAAGAGTATCA<br>TCTTTCaaaTTCTTTCCTTATACATtAGGACCTTTGCAGCATAAATT<br>ACTATACTTCTATAGACACACAAACACAAATACACACACTAAATT<br>AATA
**pGAL1**	ATATACATATCCATATCTAATCTTACTTATATGTTGTGGAAATGT<br>AAAGAGCCCCATTATCTTAGCCTAAAAAAACCTTCTCTTTGGAAC<br>TTTCAGTAATACGCTTAACTGCTCATTGCTATATTGAAGTACGGA<br>TTAGAAGCCGCCGAGCGGGTGACAGCCCTCCGAAGGAAGACTCT<br>CCTCCGTGCGTCCTCGTCTTCACCGGTCGCGTTCCTGAAACGCAG<br>ATGTGCCTCGCGCCGCACTGCTCCGAACAATAAAGATTCTACAAT<br>ACTAGCTTTTATGGTTATGAAGAGGAAAAATTGGCAGTAACCTG<br>GCCCCACAAACCTTCAAATGAACGAATCAAATTAACAACCATAGG<br>ATGATAATGCGATTAGTTTTTTAGCCTTATTTCTGGGGTAATTAA<br>TCAGCGAAGCGATGATTTTTGATCTATTAACAGATATATAAATGC<br>AAAAACTGCATAACCACTTTAACTAATACTTTCAACATTTTCGGT<br>TTGTATTACTTCTTATTCAAATGTAATAAAAGTATCAACAAAAAA<br>TTGTTAATATACCTCTATACTTTAACGTCAAGGAGAAAAAACtata

**pGPD**	CAGTTCGAGTTTATCATTATCAATACTGCCATTTCAAAGAATACG<br>TAAATAATTAATAGTAGTGATTTTCCTAACTTTATTTAGTCAAAA<br>AATTAGCCTTTTAATTCTGCTGTAACCCGTACATGCCCAAAATAG<br>GGGGCGGGTTACACAGAATATATAACATCGTAGGTGTCTGGGTG<br>AACAGTTTATTCCTGGCATCCACTAAATATAATGGAGCCCGCTTT<br>TTAAGCTGGCATCCAGAAAAAAAAAGAATCCCAGCACCAAAATAT<br>TGTTTTCTTCACCAACCATCAGTTCATAGGTCCATTCTCTTAGCG<br>CAACTACAGAGAACAGGGGCACAAACAGGCAAAAAACGGGCACA<br>ACCTCAATGGAGTGATGCAACCTGCCTGGAGTAAATGATGACAC<br>AAGGCAATTGACCCACGCATGTATCTATCTCATTTTCTTACACCT<br>TCTATTACCTTCTGCTCTCTCTGATTTGGAAAAAGCTGAAAAAAA<br>AGGTTGAAACCAGTTCCCTGAAATTATTCCCCTACTTGACTAATA<br>AGTATATAAAGACGGTAGGTATTGATTGTAATTCTGTAAATCTA<br>TTTCTTAAACTTCTTAAATTCTACTTTTATAGTTAGTCTTTTTTTT<br>TAGTTTTAAAACACCAAGAACTTAGTTTCGAATAAACACACATAA<br>ACAAACAAA
**DEG1t_pCYC1no TATA**	AATAATATATAAACCTGTATAATATAACCTTGAAGACTATATTTC<br>TTTTCttcTTTCCTTATACATtAGGACCTTTGCAGCATAAATTACTA<br>TACTTCTATAGACACACAAACACAAATACACACACTAAATTAATA
**3xlex2Op_pCYC1 min**	CAGATCCGCCAGGCGTGTATATATAGCGTGGTGCTGTATATACT<br>CACAGCATAACTGTATATACACCCAGGGATGGCCAGGCAACTTT<br>GCTGTATATACTCACAGCATAACTGTATATACACCCAGGGTAGTG<br>CTGACACATATGCTGTATATACTCACAGCATAACTGTATATACAC<br>CCAGGGCAGGCATATATATATGTGTGCGACGACACATGATCATA<br>TGGCATGCATGTGCTCTGTATGTATATAAAACTCTTGTTTTCTTC<br>TTTTCTCTAAATATTCTTTCCTTATACATTAGGACCTTTGCAGCA<br>TAAATTACTATACTTCTATAGACACACAAACACAAATACACACAC<br>TAAATTAATA

Coding regions

**yEGFPgg_GS_ssrA**	ATGTCTAAAGGTGAAGAATTATTCACTGGTGTTGTCCCAATTTTG<br>GTTGAATTAGATGGTGATGTTAATGGTCACAAATTTTCTGTCTCC<br>GGTGAAGGTGAAGGTGATGCTACTTACGGTAAATTGACCTTAAA<br>ATTTATTTGTACTACTGGTAAATTGCCAGTTCCATGGCCAACCTT<br>AGTCACTACTTTCGGTTATGGTGTTCAATGTTTTGCGAGATACCC<br>AGATCATATGAAACAACATGACTTTTTCAAGTCTGCCATGCCAGA<br>AGGTTATGTTCAAGAAAGAACTATTTTTTTCAAAGATGACGGTA<br>ACTACAAGACCAGAGCTGAAGTCAAGTTTGAAGGTGATACCTTA<br>GTTAATAGAATCGAATTAAAAGGTATTGATTTTAAAGAAGATGG<br>TAACATTTTAGGTCACAAATTGGAATACAACTATAACTCTCACAA<br>TGTTTACATCATGGCTGACAAACAAAAGAATGGTATCAAAGTTA<br>ACTTCAAAATTAGACACAACATTGAAGATGGTTCTGTTCAATTAG<br>CTGACCATTATCAACAAAATACTCCAATTGGTGATGGTCCAGTCT<br>TGTTACCAGACAACCATTACTTATCCACTCAATCTGCCTTATCCA<br>AAGATCCAAACGAAAAGAGgGACCACATGGTCTTGTTAGAATTTG<br>TTACTGCTGCTGGTATTACCCATGGTATGGATGAATTGTACAAaG<br>GTTCTgctgctaacgacgaaaactacgctctggctgctTAA

**HIStag-GS-yoClp X**	ATGaGCCATCATCATCATCATCACAGcGGTTCtACAGATAAACGCA<br>AAGATGGCTCAGGCAAATTGCTGTATTGCTCTTTTTGCGGCAAA<br>AGCCAGCATGAAGTGCGCAAGCTGATTGCCGGTCCATCCGTGTA<br>TATCTGCGACGAATGTGTTGATTTATGTAACGACATCATTCGCGA<br>AGAGATTAAAGAAGTTGCACCGCATCGTGAACGCAGTGCGCTAC<br>CGACGCCGCATGAAATTCGCAACCACCTGGACGATTACGTTATCG<br>GCCAGGAACAGGCGAAAAAAGTGCTGGCGGTCGCGGTATACAAC<br>CATTACAAAAGATTGCGCAACGGCGATACCAGCAATGGCGTCGA<br>GTTGGGCAAAAGTAACATTCTGCTGATCGGTCCGACCGGTTCCG<br>GTAAAACGCTGCTGGCTGAAACGCTGGCTAGATTGCTGGATGTT<br>CCGTTCACCATGGCCGACGCGACTACACTGACCGAAGCCGGTTAT<br>GTGGGTGAAGACGTTGAAAACATCATTCAGAAGCTGTTGCAGAA<br>ATGCGACTACGATGTCCAGAAAGCACAGCGTGGTATTGTCTACA<br>TCGATGAAATCGACAAGATTTCTCGTAAGTCAGACAACCCGTCCA<br>TTACCCGAGACGTTTCCGGTGAAGGCGTACAGCAGGCACTGTTG<br>AAACTGATCGAAGGTACGGTAGCTGCTGTTCCACCGCAAGGTGG<br>GCGTAAACATCCGCAGCAGGAATTCTTGCAGGTTGATACCTCTAA<br>GATCCTGTTTATTTGTGGCGGTGCGTTTGCCGGTCTGGATAAAG<br>TGATTTCCCACCGTGTAGAAACCGGCTCCGGCATTGGTTTTGGC<br>GCGACGGTAAAAGCGAAGTCCGACAAAGCAAGCGAAGGCGAGCT<br>GCTGGCGCAGGTTGAACCGGAAGATCTGATCAAGTTTGGTCTTA<br>TCCCTGAGTTTATTGGTCGTCTGCCGGTTGTCGCAACGTTGAATG<br>AACTGAGCGAAGAAGCTCTGATTCAGATCCTCAAAGAGCCGAAA<br>AACGCCCTGACCAAGCAGTATCAGGCGCTGTTTAATCTGGAAGG<br>CGTGGATCTGGAATTCCGTGACGAGGCGCTGGATGCTATCGCTA<br>AGAAAGCGATGGCTAGAAAAACCGGTGCCCGTGGCCTGCGTTCC<br>ATCGTAGAAGCCGCACTGCTCGATACCATGTACGATCTGCCGTCC<br>ATGGAAGACGTCGAAAAAGTGGTTATCGACGAGTCGGTAATTGA<br>TGGTCAAAGCAAACCGTTGCTGATTTATGGCAAGCCGGAAGCGC<br>AACAGGCATCTGGTGAATAA

**Flagtag-GS-ClpP**	ATGgACTACAAAGACGATGACGACAAgGGTTCTtCATACAGCGGC<br>GAACGAGATAACTTTGCACCCCATATGGCGCTGGTGCCGATGGT<br>CATTGAACAGACCTCACGCGGTGAGCGCTCTTTTGATATCTATTC<br>TCGTCTACTTAAGGAACGCGTCATTTTTCTGACTGGCCAGGTTGA<br>AGACCACATGGCTAACCTGATTGTGGCGCAGATGCTGTTCCTGG<br>AAGCGGAAAACCCAGAAAAAGATATCTATCTGTACATTAACTCCC<br>CAGGCGGGGTGATCACTGCCGGGATGTCTATCTATGACACCATG<br>CAGTTTATCAAGCCTGATGTCAGCACCATCTGTATGGGCCAGGC<br>GGCCTCGATGGGCGCTTTCTTGCTGACCGCAGGGGCAAAAGGTA<br>AACGTTTTTGCCTGCCGAATTCGCGCGTGATGATTCACCAACCGT<br>TGGGCGGCTACCAGGGCCAGGCGACCGATATCGAAATTCATGCC<br>CGTGAAATTCTGAAAGTTAAAGGGCGCATGAATGAACTTATGGC<br>GCTTCATACGGGTCAATCATTAGAACAGATTGAACGTGATACCG<br>AGCGCGATCGCTTCCTTTCCGCCCCTGAAGCGGTGGAATACGGT<br>CTGGTCGATTCGATTCTGACCCATCGTAATTGA

**LexA-HBD(hER)-VP64**	ATGAAAGCGTTAACGGCCAGGCAACAAGAGGTGTTTGATCTCAT<br>CCGTGATCACATCAGCCAGACAGGTATGCCGCCGACGCGTGCGG<br>AAATCGCGCAGCGTTTGGGGTTCCGTTCCCCAAACGCGGCTGAA<br>GAACATCTGAAGGCGCTGGCACGCAAAGGCGTTATTGAAATTGT<br>TTCCGGCGCATCACGCGGGATTCGTCTGTTGCAGGAAGAGGAAG<br>AAGGGTTGCCGCTGGTAGGTCGTGTGGCTGCCGGTGAACCACTT<br>CTGGCGCAACAGCATATTGAAGGTCATTATCAGGTCGATCCTTC<br>CTTATTCAAGCCGAATGCTGATTTCCTGCTGCGCGTCAGCGGGA<br>TGTCGATGAAAGATATCGGCATTATGGATGGTGACTTGCTGGCA<br>GTGCATAAAACTCAGGATGTACGTAACGGTCAGGTCGTTGTCGC<br>ACGTATTGATGACGAAGTTACCGTTAAGCGCCTGAAAAAACAGG<br>GCAATAAAGTCGAACTGTTGCCAGAAAATAGCGAGTTTAAACCA<br>ATTGTCGTTGACCTTCGTCAGCAGAGCTTCACCATTGAAGGGCT<br>GGCGGTTGGGGTTATTCGCAACGGCGACTGGCTGTCATCTGCTG<br>GAGACATGAGAGCTGCCAACCTTTGGCCAAGCCCGCTCATGATC<br>AAACGCTCTAAGAAGAACAGCCTGGCCTTGTCCCTGACGGCCGA<br>CCAGATGGTCAGTGCCTTGTTGGATGCTGAGCCCCCCATACTCTA<br>TTCCGAGTATGATCCTACCAGACCCTTCAGTGAAGCTTCGATGAT<br>GGGCTTACTGACCAACCTGGCAGACAGGGAGCTGGTTCACATGA<br>TCAACTGGGCGAAGAGGGTGCCAGGCTTTGTGGATTTGACCCTC<br>CATGATCAGGTCCACCTTCTAGAATGTGCCTGGCTAGAGATCCTG<br>ATGATTGGTCTCGTCTGGCGCTCCATGGAGCACCCAGTGAAGCT<br>ACTGTTTGCTCCTAACTTGCTCTTGGACAGGAACCAGGGAAAAT<br>GTGTAGAGGGCATGGTGGAGATCTTCGACATGCTGCTGGCTACA<br>TCATCTCGGTTCCGCATGATGAATCTGCAGGGAGAGGAGTTTGT<br>GTGCCTCAAATCTATTATTTTGCTTAATTCTGGAGTGTACACATT<br>TCTGTCCAGCACCCTGAAGTCTCTGGAAGAGAAGGACCATATCCA<br>CCGAGTCCTGGACAAGATCACAGACACTTTGATCCACCTGATGGC<br>CAAGGCAGGCCTGACCCTGCAGCAGCAGCACCAGCGGCTGGCCC<br>AGCTCCTCCTCATCCTCTCCCACATCAGGCACATGAGTAACAAAG<br>GCATGGAGCATCTGTACAGCATGAAGTGCAAGAACGTGGTGCCC<br>CTCTATGACCTGCTGCTGGAGATGCTGGACGCCCACCGCCTACAT<br>GCGCCCACTAGCCGTGGAGGGGCATCCGTGGAGGAGACGGACCA<br>AAGCCACTTGGCCACTGCGGGCTCTACTTCATCGGACGCGCTGG<br>ACGATTTCGATCTCGACATGCTGGGTTCTGATGCCCTCGATGACT<br>TTGACCTGGATATGTTGGGAAGCGACGCATTGGATGACTTTGAT<br>CTGGACATGCTCGGCTCCGATGCTCTGGACGATTTCGATCTCGAT<br>ATGTTATAA

Terminators

**CYC1t**	CATGTAATTAGTTATGTCACGCTTACATTCACGCCCTCCCCCCAC<br>ATCCGCTCTAACCGAAAAGGAAGGAGTTAGACAACCTGAAGTCT<br>AGGTCCCTATTTATTTTTTTATAGTTATGTTAGTATTAAGAACGT<br>TATTTATATTTCAAATTTTTCTTTTTTTTCTGTACAGACGCGTGT<br>ACGCATGTAACATTATACTGAAAACCTTGCTTGAGAAGGTTTTG<br>GGACGCTCGAAGGCTTTAATTTGCAAGCTatc
**Tsynth15**	TATATAACTGTCTAGAAATAAAGAGTATCATCCAA
**Tsynth16**	TATATAACTGTCTAGAAATAAAGAGTATCATCCAAAAA

References

[1] Marchisio, M. A. (2014). In silico design and in vivo implementation of yeast gene Boolean gates. Journal of Biological Engineering, 8(1), 6.

[2] Marchisio, M. A. (2021) Modular Modeling of Genetic Circuits in SBML Level 3. Methods in Molecular Biology Vol. 2189(17), 45-63: Computational Methods in Synthetic Biology, 2nd Edition. MA Marchisio Editor, Springer-Verlag, New York.

[3] Grilly, C., Stricker, J., Pang, W. L., Bennett, M. R., and Hasty, J. (2007). A synthetic gene network for tuning protein degradation in Saccharomyces cerevisiae. Molecular Systems Biology, 3(1), 127.

[4] Curran, K. A., Morse, N. J., Markham, K. A., Wagman, A. M., Gupta, A., and Alper, H. S. (2015). Short Synthetic Terminators for Improved Heterologous Gene Expression in Yeast. ACS Synthetic Biology, 4(7), 824?832.
